# Biomechanical Effects of a Passive Lower-Limb Exoskeleton Designed for Half-Sitting Work Support on Walking

**DOI:** 10.3390/s25164999

**Published:** 2025-08-12

**Authors:** Qian Li, Naoto Haraguchi, Bian Yoshimura, Sentong Wang, Makoto Yoshida, Kazunori Hase

**Affiliations:** 1 Department of Mechanical Systems Engineering, Tokyo Metropolitan University, Tokyo 191-0065, Japan; haraguchi-naoto@rehab.go.jp (N.H.); yoshimura.bian@gmail.com (B.Y.); wang-sentong@hirosaki-u.ac.jp (S.W.); yoshida-makoto@tmu.ac.jp (M.Y.); 2 Department of Assistive Technology, Research Institute of National Rehabilitation Center for Persons with Disabilities, Tokorozawa 359-8555, Japan; 3 Department of Mechanical Science and Engineering, Hirosaki University, Aomori 036-8560, Japan

**Keywords:** passive lower-limb exoskeleton, wearable chair, biomechanical analysis, inverse dynamics, gait, force sensors, motion capture, contact forces, contact moments

## Abstract

The half-sitting posture is essential for many functional tasks performed by industrial workers. Thus, passive lower-limb exoskeletons, known as wearable chairs, are increasingly used to relieve lower-limb loading in such scenarios. However, although these devices lighten muscle effort during half-sitting tasks, they can disrupt walking mechanics and balance. Moreover, rigorous biomechanical data on joint moments and contact forces during walking with such a device remain scarce. Therefore, this study conducted a biomechanical evaluation of level walking with a wearable chair to quantify its effects on gait and joint loading. Participants performed walking experiments with and without the wearable chair. An optical motion capture system and force plates collected kinematic and ground reaction data. Six-axis force sensors measured contact forces and moments. These measurements were fed into a Newton–Euler inverse dynamics model to estimate lower-limb joint moments and assess joint loading. The contact measurements showed that nearly all rotational load was absorbed at the thigh attachment, while the ankle attachment served mainly as a positional guide with minimal moment transfer. The inverse dynamics analysis revealed that the wearable chair introduced unintended rotational stresses at lower-limb joints, potentially elevating musculoskeletal risk. This detailed biomechanical evidence underpins targeted design refinements to redistribute loads and better protect lower-limb joints.

## 1. Introduction

In industrial production and warehousing, workers frequently adopt a half-sitting posture for extended, repetitive tasks such as assembly, drilling, and material handling [1,2]. This posture positions the torso and upper limbs at an ergonomic height while avoiding the greater metabolic cost and space requirements of squatting or kneeling [3]. While this posture partially relieves spinal loading, it also can exacerbate muscle fatigue, produces uneven joint loading, and elevates the risk of musculoskeletal disorders [4]. To address these problems, battery-free passive exoskeletons have emerged as an effective solution [5,6,7,8]. Among these, the wearable chair is considered particularly suitable for half-sitting environments [9]. When the wearer lowers into a half-sitting position, the bottom of the device contacts the ground and supports the wearer’s weight, thereby reducing the load on the lower limbs. Once the wearer stands fully upright, the device lifts clear off the floor, allowing normal walking and other movements [10,11,12].

In real industrial scenarios, workers wearing wearable chairs must frequently alternate between functional tasks and walking between different workstations [12]. Previous studies on wearable chairs during half-sitting tasks have demonstrated the chairs’ ability to significantly reduce lower-limb muscle activity, thereby effectively delaying fatigue onset and potentially enhancing task endurance [13,14]. However, exoskeletons frequently reduce the load during the intended task while affecting performance in other tasks [15]. These effects raise concerns regarding potential safety risks and ergonomic challenges associated with the practical use of wearable chairs beyond functional task environments [16]. A previous study further noted that during non-functional tasks, particularly level walking, wearable chairs significantly alter gait parameters, compromise balance, and heighten subjective discomfort [17]. Although such findings provide an initial glimpse of their effects on walking performance, the existing evidence is largely confined to gait measurements and subjective assessments. Systematic biomechanical analyses of gait characteristics, joint kinetics, and lower-limb contact forces during walking with wearable chairs remain scarce. Notably, no study has yet quantified three-dimensional (3D) hip, knee, and ankle joint moments in this context. Moreover, prolonged alterations in gait patterns and balance may increase the risk of cumulative musculoskeletal injuries or accidental falls [18,19], emphasizing the critical need for detailed biomechanical analyses.

Therefore, this study aimed to conduct a comprehensive biomechanical analysis of walking while wearing a wearable chair, utilizing external force measurements, 3D motion capture, and inverse dynamics methodologies to quantify its effects on gait and joint loading. By systematically evaluating these biomechanical outcomes, this study clarifies both the load reduction benefits and potential drawbacks associated with wearable chairs in realistic industrial settings. Ultimately, these insights aim to inform future exoskeleton design improvements, facilitate their safe industrial implementation, and establish guidelines that promote user health, comfort, and productivity.

## 2. Materials and Methods

### 2.1. Participants

The participants comprised 11 healthy males (height: 1.73 ± 0.05 m; weight: 63.2 ± 5.5 kg; age: 22.9 ± 1.7 years). All participants had no history of neurological, muscular, or skeletal disorders affecting walking. This study received approval from the Ethics Committee of Tokyo Metropolitan University (protocol code R6-001, 19 April 2024). Before the experiment, all participants received verbal and written explanations of the study’s content and provided written informed consent.

### 2.2. Equipment

**Figure 1:**
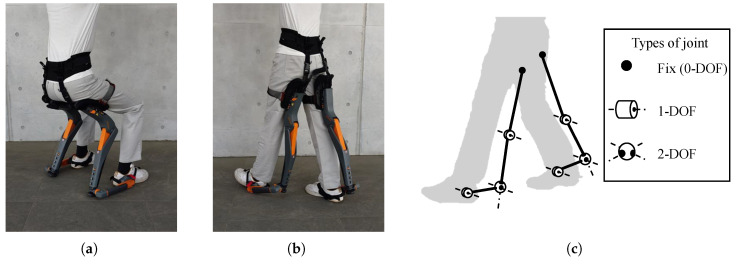
This figure illustrates the wearable chair (ChairlessChair; noonee AG, Switzerland) in two use case scenarios and depicts its joint architecture. (**a**) A half-sitting scenario. (**b**) A walking scenario. (**c**) The degrees of freedom (DOFs) of the joints in the wearable chair.

The wearable chair device utilized in this study was a passive lower-limb exoskeleton (ChairlessChair; noonee AG, Hedingen, Switzerland) designed specifically to support half-sitting postures and short-distance movements frequently encountered in industrial or occupational settings. As shown in Figure 1, it comprises rigid supports mounted along the thighs and ankles, allowing the effective transmission and distribution of body weight.

Kinematic data were recorded using an optical motion capture system (OptiTrack Flex3; Natural Point Inc., Corvallis, OR, USA). Ground reaction forces (GRFs) were captured with two embedded force plates (9286AA; Kistler Group, Winterthur, Switzerland). Contact forces and moments at the interfaces between a participant and the wearable chair were measured with high-precision six-axis force sensors mounted at the thighs (USX10-H10-500N; Tec Gihan Co., Ltd., Kyoto, Japan) and ankles (FFS080YS102U6; Leptrino Co., Ltd., Nagano, Japan).

### 2.3. Experimental Conditions

Participants performed level walking under two conditions: with and without the wearable chair. Each trial began from a stationary neutral standing position. To emulate the wearable chair’s actual usage scenario, in which it is typically engaged only for the half-sitting tasks rather than prolonged walking, the analysis intentionally focused on the very first gait cycle. This cycle was selected because it involves the highest biomechanical load, reflecting the demands of starting to walk from standing still. Participants walked at a comfortable self-selected speed, with multiple practice attempts ensuring consistent stepping on force plates placed approximately 0.7 m apart. Three trials were recorded per condition, selecting the trial that best represented natural gait for further analysis. Conditions were randomized to mitigate fatigue and order effects.

### 2.4. Measurements

**Figure 2:**
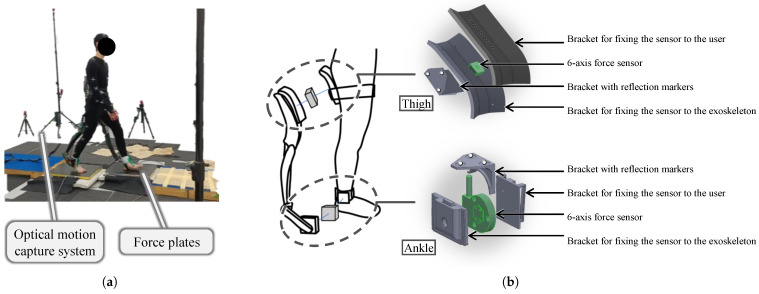
(**a**) Measurements of kinematic data and GRFs during the walking experiment using an optical motion capture system and force plates. (**b**) Measurement of the contact forces between the human body and the wearable chair at the thigh and ankle of the right leg. A force sensor was placed between two brackets; one bracket was attached to the user’s body, and the other to the wearable chair. The position and posture of the sensor were measured with reflective markers on an additional bracket. Brackets and sensors were placed on the left leg in the same way.

Each participant’s height and weight were measured prior to the experiments. Kinematic data were captured using an optical motion capture system, as shown in Figure 2a. Eighteen reflective markers were attached to anatomical landmarks on participants’ lower bodies and shoulders, conforming to the Plug-in Gait model [20]. The marker data were digitally filtered using a low-pass filter (Butterworth fourth-order type, −3 dB at 6 Hz) and were sampled at 100 Hz. The 6 Hz cut-off was selected after residual analysis of representative trials, which showed that more than 95% of the kinematic signal power lay below this frequency [21]. Identical settings are widely adopted in contemporary gait analysis studies [22,23]. GRFs were captured at 1000 Hz with two embedded force plates, as shown in Figure 2a. Additionally, contact forces and moments were measured using six-axis force sensors mounted bilaterally at the thigh (sampled at 1000 Hz) and ankle (sampled at 1200 Hz) regions. As illustrated in Figure 2b, custom-designed polylactic acid brackets secured the sensors to the wearable chair, ensuring accurate force transmission without extraneous contacts. Sensor positioning was validated in pilot tests, which confirmed stable mounting and highly consistent force measurements across repeated trials. Reflective markers on sensor brackets facilitated coordinate system transformations between sensor and global coordinate systems, allowing precise localization and orientation calculations. GRFs and sensor data were filtered using a low-pass filter (Butterworth fourth-order type, −3 dB at 18 Hz). A cut-off frequency of 18 Hz was selected because walking GRF signals show almost no meaningful content above 20 Hz [24]. An identical 18 Hz fourth-order Butterworth filter has been adopted in recent studies [10,22].

### 2.5. Data Analysis

#### 2.5.1. Gait Parameters

Gait cycle duration, stride length, and walking speed were extracted from motion capture and force plate data. The gait cycle was defined as the interval between consecutive right heel contacts. Stride length was calculated based on right heel marker displacement over the gait cycle, and walking speed was determined by dividing stride length by gait cycle duration.

#### 2.5.2. Inverse Dynamics

**Figure 3:**
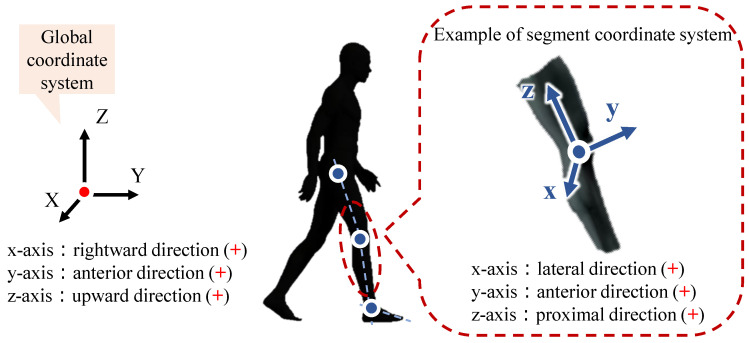
The coordinate systems used for the inverse dynamics calculations were defined as follows. In the global frame, the +x axis points rightward; the +y axis, anteriorly; and the +z axis, upward. In each segment’s local frame, the +x axis points laterally; the +y axis, anteriorly; and the +z axis, proximally along the segment. Although the axis labels differ from the recommended anatomical sequence of the International Society of Biomechanics (ISB) [26,27,28], the definition is still fully ISB-compliant because simple cyclic relabeling aligns the axes with the ISB convention.

Inverse dynamics analysis was conducted using human movement analysis software (JoDyn; HumTec Co., Ltd., Tokoyo, Japan), which integrates kinematic data from the motion capture system and external force data obtained from the force plates and the six-axis force sensors [25]. The software built a rigid-body, segmental model of the human body. The segmental center of mass and joint center coordinates were calculated from the marker position data. These data were used as the basis for inverse dynamics analysis via the Newton–Euler equations, and joint reaction forces and moments along three orthogonal axes were obtained, preserving full 3D dynamics. Figure 3 illustrates the global and segment coordinate systems used in these calculations.

Firstly, the joint reaction forces $F_{x}^{s},F_{y}^{s}$, $F_{z}^{s}$ for the *s*-th segment were calculated as follows:

(1) FxsFysFzs=msaxsaysazs+Fxs−1Fys−1Fzs−1+Rsgloseg00msg−Fext_xssegFext_yssegFext_zsseg

where $m^{s}$ is the mass; $a_{x}^{s}$, $a_{y}^{s}$, and $a_{z}^{s}$ represent the linear accelerations of the segment’s center of mass; $F_{x}^{s-1}$, $F_{y}^{s-1}$, and $F_{z}^{s-1}$ are joint reaction forces from the distal adjacent segment; Rsgloseg is the rotation matrix transforming forces from the global coordinate system to the segment coordinate system; *g* is gravitational acceleration; and Fext_xsseg, Fext_ysseg, and Fext_zsseg are external forces, expressed in the segment coordinate system.

Subsequently, joint moments $M_{x}^{s}$, $M_{y}^{s}$, and $M_{z}^{s}$ for the *s*-th segment were calculated based on Newton–Euler equations using the segment’s center of mass as the origin:

(2) Mxs=Ixsαxs+(Izs−Iys)ωysωzs+Fys−1lds+Fyslps+Mxs−1−Mext_xsseg

(3) Mys=Iysαys+(Ixs−Izs)ωzsωxs−Fxs−1lds−Fxslps+Mys−1−Mext_ysseg

(4) Mzs=Izsαzs+(Iys−Ixs)ωxsωys+Mzs−1−Mext_zsseg

where $I_{x}^{s}$, $I_{y}^{s}$ and $I_{z}^{s}$ are the principal moments of inertia around the segment’s local *x*, *y*, and *z* axes, respectively; $\alpha_{x}^{s}$, $\alpha_{y}^{s}$, and $\alpha_{z}^{s}$ are the angular accelerations about the local segment axes; $\omega_{x}^{s}$, $\omega_{y}^{s}$, and $\omega_{z}^{s}$ are the angular velocities about these axes; $l_{d}^{s}$ and $l_{p}^{s}$ are the distances from the center of mass to the distal and proximal joints, respectively; $M_{x}^{s-1}$, $M_{y}^{s-1}$, and $M_{z}^{s-1}$ are joint moments from the distal segment; and Mext_xsseg, Mext_ysseg, and Mext_zsseg are the external moments in the segment coordinate system.

Finally, the calculated joint reaction forces and moments were normalized by each participant’s mass to facilitate comparisons across participants. Although normalization by both body mass and leg length is possible [29], only mass-normalized values are reported because the participants’ heights were similar. This normalization is common in other studies [21,22].

Additionally, because the external forces and moments were recorded with force sensors and force plates in this study, the measurements were expressed in each sensor’s local coordinate system. Prior to the inverse dynamics computation, these data were transformed into the required coordinate system using the rotation matrices obtained from the motion capture data of the reflective marker attached to the sensors. The explicit transformation formulas and notation are provided in Appendix A.

#### 2.5.3. Peak Moments

Peak moment is a widely used parameter in gait analysis, defined as the maximum absolute value of the joint moment occurring within each gait cycle. It provides insights into the maximal biomechanical demands placed on the joints during walking [30]. In this study, peak moments were calculated separately for the hip, knee, and ankle joints in three anatomical planes.

Firstly, the joint moment vector $M^{s}\left(t\right)$ for the *s*-th segment at time *t* is defined as

(5) $$M^{s}\left(t\right)=\left[\begin{array}{c} M_{x}^{s}\left(t\right)\\ M_{y}^{s}\left(t\right)\\ M_{z}^{s}\left(t\right) \end{array}\right]$$

where $M_{x}^{s}\left(t\right)$, $M_{y}^{s}\left(t\right)$, and $M_{z}^{s}\left(t\right)$ are the instantaneous joint moment components calculated using Equations (2)–(4).

Subsequently, the peak moments for each plane are computed as follows:

(6) $$M_{peak\_x}^{s}=\max_{t}\left|M_{x}^{s}\left(t\right)\right|$$

(7) $$M_{peak\_y}^{s}=\max_{t}\left|M_{y}^{s}\left(t\right)\right|$$

(8) $$M_{peak\_z}^{s}=\max_{t}\left|M_{z}^{s}\left(t\right)\right|$$

Finally, all peak moment values were normalized to each body mass and stride length to facilitate comparisons across participants. In contrast to Section 2.5.2, where moments were normalized only by each participant’s mass, the present analysis further divides these values by stride length. This additional scaling is warranted because experimental evidence shows that excessive over-striding significantly increases peak moments far more than it affects the overall mean level of the moment time series curve [31]. Two-stage normalization therefore reduces this confounding influence and preserves the statistical robustness of the peak moment metrics [32].

### 2.6. Statistics

In this study, statistical analysis was performed on gait parameters and peak moments. The normality of all data was assessed using the Shapiro–Wilk test. Variables satisfying normal distribution assumptions were compared between the two experimental conditions (with and without the wearable chair) using two-tailed paired *t*-tests, and paired-samples Cohen’s *d* was calculated as an effect size. Otherwise, the Wilcoxon signed-rank test was employed, and effect size *r* was obtained from the test statistic. Statistical significance was set at p<0.05 (significant) and p<0.01 (highly significant). MATLAB R2022a (MathWorks Inc., Natick, MA, USA) software was used to perform all data analyses and visualizations. All data are presented as means ± standard deviation (SD).

## 3. Results

### 3.1. Gait Parameters

Table 1 summarizes the statistically significant differences in gait parameters between walking with and without the wearable chair. Wearing the device lengthens gait cycle duration by 12.4% (Cohen’s d=1.70), while reducing stride length by 5.1% (Cohen’s d=0.99) and walking speed by 15.6% (Cohen’s d=1.79), indicating that the wearable chair influences both temporal and spatial aspects of gait.

### 3.2. Contact Force and Moment

**Figure 4:**
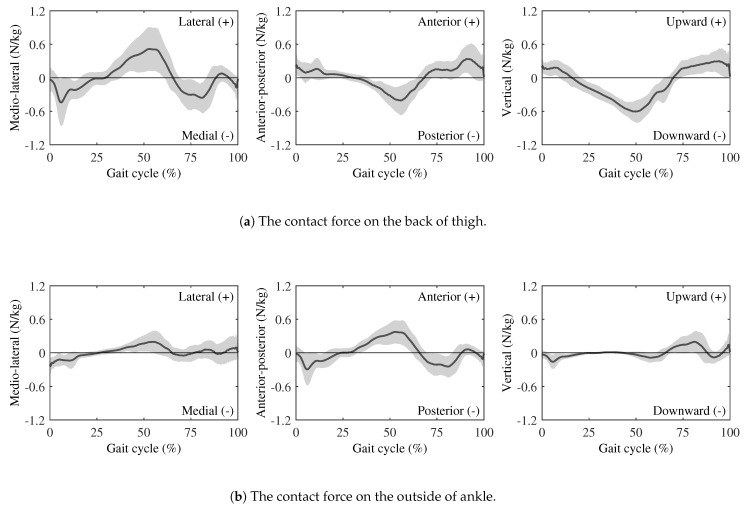
Contact forces are measured by force sensors. The axes follow the global coordinate system in Figure 3. The solid curves and the gray shading indicate the average and SD, respectively. All force magnitudes are normalized to each participant’s mass.

**Figure 5:**
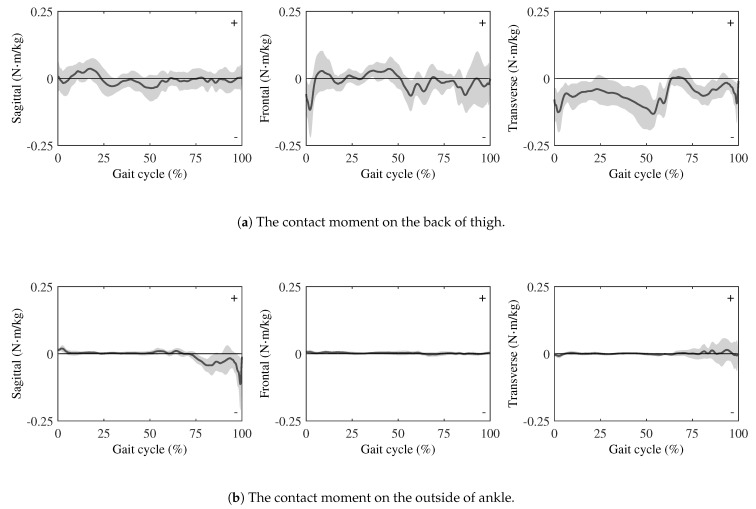
Contact moments are measured by force sensors. The directions of these moments follow the right-hand rule in the global coordinate system of Figure 3. The solid curves and the gray shading indicate the average and SD, respectively. All moment magnitudes are normalized to each participant’s mass.

Figure 4 and Figure 5 present the contact force and moment curves between the human body and the wearable chair over one gait cycle. Raw signals from the force sensors were transformed into the global coordinate system using the motion capture’s rotation matrices, thereby representing forces acting on the body.

**The results of the contact force**

As shown in Figure 4a, at the thigh, the wearable chair imposes modest, gait-synchronized forces:

- At the medio-lateral interface, the device initially pushed medially, then reversed direction, and rose steadily to a peak lateral force near mid-stance, before declining toward zero during late swing.
- At the anterior–posterior interface, a posterior-directed force emerged in early stance and reached a peak posterior force near mid-stance. The direction switched to anterior around three-quarters of the gait cycle, peaking just during terminal stance.
- At the vertical interface, downward loading dominated mid-stance, followed by an upward rebound during terminal stance.

As shown in Figure 4b, at the ankle, all force components were markedly smaller than those at the thigh:

- The medio-lateral trace showed a shallow biphasic pattern similar in shape to that at thigh but with about half the amplitude;
- Conversely, the anterior–posterior trace displayed an inverted profile, peaking anteriorly at mid-stance and remaining close to zero through terminal stance and swing;
- Vertical forces remained close to zero.

**The results of the contact moment**

As shown in Figure 5a, at the thigh, the moments transmitted through the wearable chair were small in all planes:

- In the sagittal plane, a brief negative deflection appeared just after heel strike, followed by a shallow positive peak. The curve then oscillated gently about zero during mid-stance and swing.
- In the frontal plane, a brief negative deflection was shown immediately after heel strike, followed by a small positive excursion in early stance. It then remained near zero with low-amplitude oscillations, exhibited a slight negative bias from mid-stance to terminal stance, and returned toward zero during swing.
- In the transverse plane, the component remained negative through most of stance, rose sharply around mid-stance and exhibited a brief positive excursion, then stayed slightly negative during terminal stance.

By contrast, the ankle attachment conveyed practically no moment, as shown in Figure 5b. Across the entire gait cycle, the sagittal, frontal, and transverse moments hovered near zero, with only a slight change in the last quarter of the gait cycle.

### 3.3. Joint Moments

**Figure 6:**
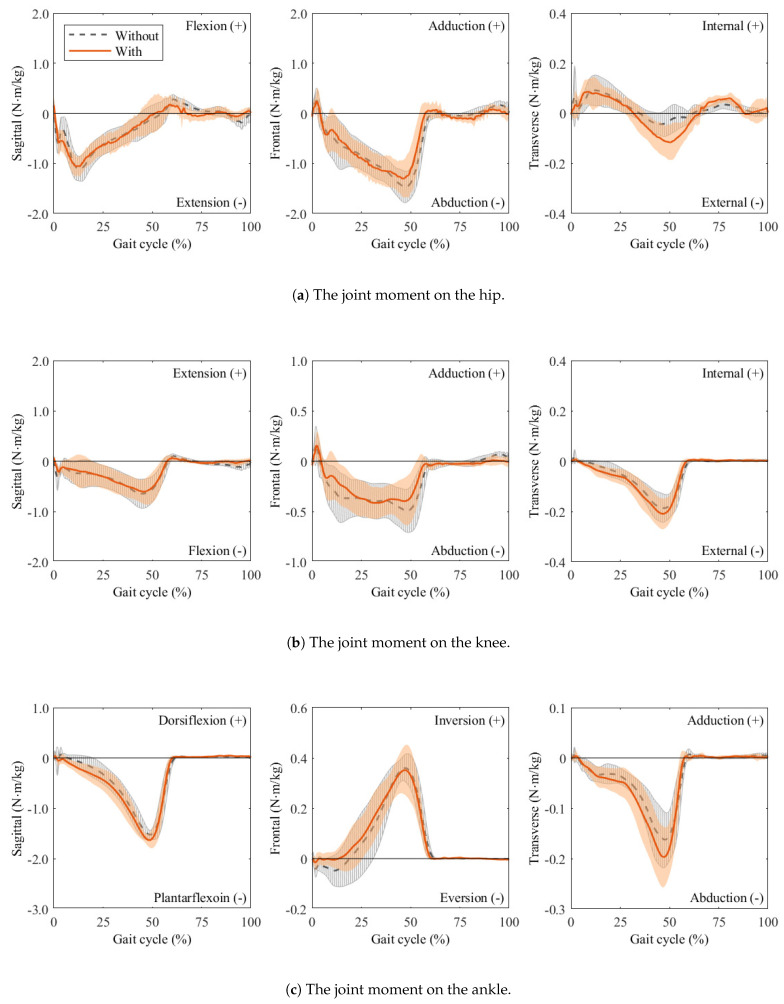
The joint moment curves are computed using inverse dynamics. The gray dashed curves with hatched bands indicate the mean ± SD for walking without the wearable chair, whereas the red solid curves with shaded bands indicate those for walking with the wearable chair. All moment magnitudes are normalized to each participant’s mass.

**Figure 7:**
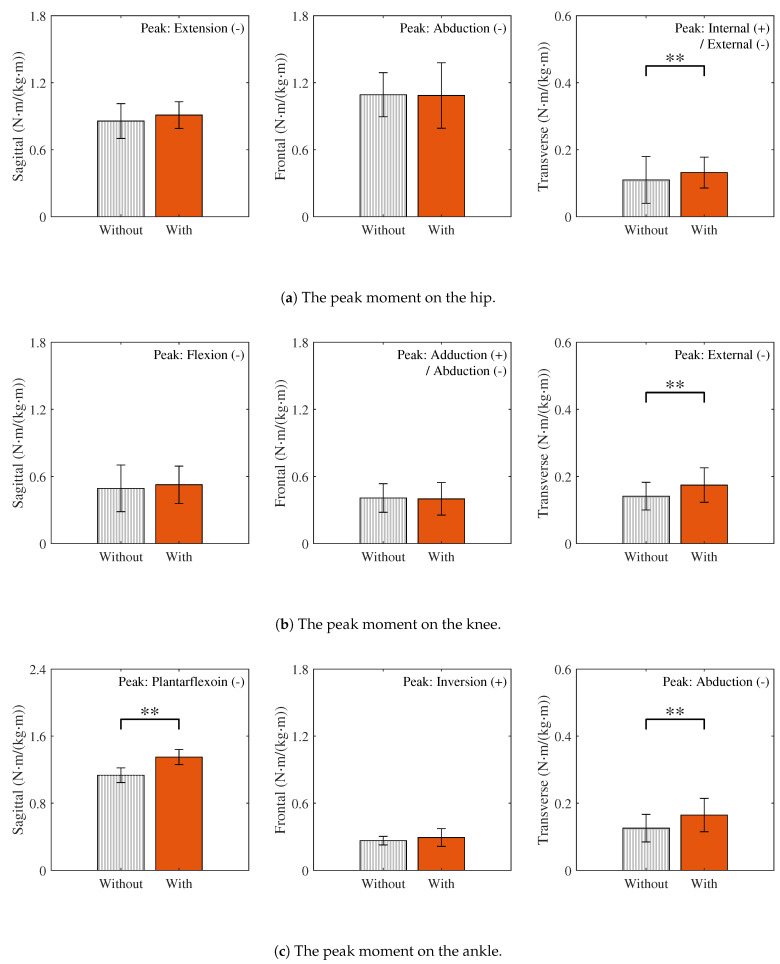
The peak moments with the direction of the peak indicated in every panel. The bars and error bars represent the average and SD, respectively. All values are normalized to the mass and stride length of participants. Statistically significant differences between walking with and without the wearable chair are marked with ** p<0.01.

Figure 6 presents the joint moment curves over one gait cycle, computed using inverse dynamics, for walking with and without the wearable chair. Peak moments are summarized in Figure 7. For joints whose peaks occur in the negative direction, the absolute values of those peaks are reported and the original direction is indicated. Because the directions of the knee adduction and hip internal rotation peaks vary across participants, both directions are shown.

**Sagittal plane:** The hip flexion, knee extension, and ankle dorsiflexion moments, which are primarily responsible for sagittal plane kinetics, mainly represent the generation and absorption of forward propulsion. Among these, the peak moment at the ankle joint increased significantly with the wearable chair and exhibited a large effect size (r=1.00). In contrast, no statistically significant increase was observed at the hip and knee joints. The corresponding effect sizes at these joints were small (hip: Cohen’s d=0.29; knee: Cohen’s d=0.26).
**Frontal plane:** The hip adduction, knee adduction, and ankle inversion moments, which are primarily responsible for frontal plane kinetics, mainly represent control of lateral balance and support against gravity. Although the additional weight of the wearable chair was expected to increase the gravitational load and thus these peak moments, no statistically significant differences were observed between walking with and without the wearable chair. Moreover, the corresponding effect sizes were also small (hip: Cohen’s d=0.04; knee: Cohen’s d=0.09; ankle: r=0.30).
**Transverse plane:** The hip internal rotation, knee internal rotation, and ankle adduction moments, which are primarily responsible for transverse plane kinetics, mainly represent the rotational control of the body. The peak moments at all these joints showed statistically significant increases with the wearable chair. Moreover, the corresponding effect sizes were also large (hip: r=0.56; knee: Cohen’s d=1.59; ankle: Cohen’s d=1.71).

## 4. Discussion

### 4.1. Reliability of Experimental Results

The reliability of biomechanical measurements is essential for accurately interpreting the effects of wearable chairs. The results of the gait parameters are consistent with their changes observed in evaluations with another wearable chair [17]. This suggests that they are typical responses to the additional load and mechanical constraints of wearable chairs. This consistency substantiates the validity of our gait parameter measurements. Table 1 indicates the prolonged gait cycle and shortened stride length, likely reflecting an adaptive strategy to maintain balance and stability when wearing the wearable chair. In addition, the mechanical load and inertial effects introduced by the device may have led users to adopt a more cautious walking pattern, reducing walking speed as a trade-off to ensure safe and controlled movement.

In addition, accurate biomechanical analysis is essential for reliably evaluating the effects of wearable chairs. Kadaba [33] reported excellent intra-subject repeatability, with large coefficient of multiple correlation (CMC) values across three gait trials. These results indicate that a single gait evaluation can obtain data that are reliable enough. The joint moment curves shown in Figure 6 are almost consistent with the typical gait patterns reported in the previous study [21], both with and without the wearable chair. Minor deviations were observed in our study, such as the absence of early stance knee extension moments. These are due to the analysis of the first gait cycle rather than calculation errors, thereby confirming the integrity of our joint moment measurements and calculations.

### 4.2. Evaluation of Wearable Chair Effects on Lower-Limb Joint Loads

The contact forces and moments measured at the thigh and ankle attachments provide direct insight into how the wearable chair alters lower-limb loading during gait. Many previous studies used a spring–damper model to express the human-exoskeleton interaction model [34,35]. Figure 4a shows that the three force components at the thigh exhibit a spring–damper-like behavior; during early stance, the device exerts downward, backward, and medial forces, which then reverse during the swing phase. This force pattern reflects a cyclic push–pull interaction between the wearable chair and the user’s thigh. Figure 5a demonstrates that the moments in all anatomical planes also capture the dynamic interaction between the wearable chair and the user’s lower-limb during walking. In addition, to mitigate shear and torsional loads at the human–exoskeleton interface, most lower-limb exoskeletons incorporate multiple passive DOFs [36]. The wearable chair includes a distal two-DOF joint at the ankle, which can act as a passive joint, as shown in Figure 1c. Figure 4b shows that the forces measured at the ankle are substantially smaller, suggesting that the distal two-DOF joint decouples most of the load from the shank and foot. As shown in Figure 5b, a similar trend is observed in the joint moments, which are negligible at the ankle. These results indicate that the distal joints effectively isolate the ankle, allowing the ankle attachment to serve primarily as a positional guide rather than a torque-transmitting interface. Overall, these findings indicate that rotational coupling between the wearable chair and the user is concentrated at the proximal thigh attachment, whereas the distal joint protects the ankle from appreciable moment transfer.

Although external loading at the ankle was small, inverse dynamics analysis revealed significant increases in hip and knee internal rotation moments and in ankle adduction and dorsiflexion moments when the wearable chair was worn, as shown in Figure 6 and Figure 7. The remaining joint moments showed no significant changes, indicating that the wearable chair does not affect all joints uniformly. Plane-specific analyses clarify these observations. In the sagittal planes, the significant increase in the ankle dorsiflexion moment can be attributed to the additional force required for push-off due to the extra weight of the wearable chair. As ankle plantarflexion normally produces the largest moment during gait [37,38], it is especially sensitive to this additional demand. Moreover, all three joints did not change significantly in the frontal planes. In contrast, all three joints exhibited significantly larger moments in the transverse planes. These differences can be attributed to contact forces at the medio-lateral interface, which directly affect joint moments in both the transverse and frontal planes. However, because joint moments in the transverse plane are relatively smaller than those in the frontal plane, they are more susceptible to the influence of these medio-lateral contact forces. Consequently, the lateral sway induced by the wearable chair significantly exacerbates joint moment alterations in the transverse planes.

### 4.3. Risks from Altered Joint Loads and Design Implications

A previous study examined the effect of a wearable chair on gait parameters, balance, and subjective discomfort [17]. Other previous studies on lower-limb load carriage in adults have similarly reported alterations in gait parameters and the increased activation of lower-limb muscles [39,40]. By comparison, the dynamic analysis method employed in this study effectively identified critical biomechanical impacts of the wearable chair on the anatomical planes using measurements of contact data. Particularly pronounced were the increased joint rotational loads in the transverse planes, whereas most sagittal and frontal plane moments remained unchanged. The transverse plane joint moments at the hip, knee, and ankle all remained below the 0.3 (N·m/kg ) recommended threshold reported by multiple studies [41,42,43]. Nevertheless, because rotational motion in the transverse plane at the knee and ankle is constrained largely by passive tissues rather than by musculature [44,45], even moderate rises in those moments elevate the risk of ligamentous overload and, through potential cumulative load effects during long-term use, early osteochondral degeneration [46]. Corroborating this concern, occupational health studies have shown that roofers kneeling on sloped roofs twist their knees inward by about 10–15° and experience significantly higher transverse plane moments, which are linked to meniscus injuries [47]. During asymmetric manual material-handling tasks, data from knee implants show that lifting uneven loads while twisting increases knee forces compared with straight lifts, adding extra stress on the ligaments [48]. This means that the effects of the wearable chair in the transverse planes should be given greater importance in the results of this study in terms of the risk of musculoskeletal disorders and injuries.

These effects in transverse planes highlighted the necessity for targeted design improvements. Potential improvement strategies include shortening the exoskeleton frame length to minimize lateral sway radius and incorporating transverse DOFs at the ankle attachments to reduce mechanical interference. These modifications can decrease excessive mechanical stress while preserving the device’s primary functionality for half-sitting tasks. This trade-off between reducing biomechanical load during walking and maintaining usability underscores the importance of dynamic analysis as a tool for improving exoskeleton design, thereby enhancing safety and efficacy in real-world applications.

### 4.4. Limitations and Future Work

Despite the robustness of our analysis, some limitations remain. First, focusing on the first gait cycle enhances ecological realism by capturing the transition from standing to walking. However, this sacrifices generalizability to steady-state gait mechanics and precludes evaluation of longitudinal adaptations in joint loading, namely whether the elevated transverse plane moments persist, diminish, or intensify with continued use [49,50]. Meanwhile, the abrupt psychological shift from the supported half-sitting posture to subsequent walking may have induced cautious steps, possibly biasing the measured loads [51]. Additionally, the joint moments obtained through inverse dynamics analysis included the specific contribution of muscle forces as well as other factors such as passive resistances in the tissues and mechanical responses in the body [52]. Moreover, all 11 participants were healthy males and we tested only a single prototype of the wearable chair. There is some randomness because the analysis relied on just one trial for each condition. Therefore, the results may not generalize to female users, older or injured populations, or alternative device designs. Finally, the present method requires the manufacture of components that fix the six-axis force sensors to the device for recording the contact forces. This increases the complexity of the experimental setup, which is a disadvantage of the proposed method.

To address these limitations, future studies could apply complementary biomechanical analysis methods, such as musculoskeletal modeling or electromyography [10,13], to better interpret the sources of the measured joint moments. Additionally, integrating these methods with optimization techniques may facilitate the identification of design parameters that minimize harmful joint loads and improve device safety and comfort. Furthermore, collecting and analyzing multiple consecutive gait cycles across repeated sessions would extend the analysis to steady-state walking and enable evaluation of longitudinal adaptations in joint loading. Future investigations should also recruit participants of both sexes with diverse body sizes and evaluate multiple wearable chair designs to enhance generalizability.

## 5. Conclusions

The contact forces and moments at the interfaces between the wearable chair and the body were directly measured in this study, and it was shown that most of the rotational load was concentrated at the thigh attachment, whereas the ankle was largely shielded from substantial moment transfer by the distal joints of the device. By combining these measurements with motion capture kinematics and GRFs, this study evaluated the effects of a wearable chair on the first gait cycle by focusing on joint moments obtained from inverse dynamics analysis. The results show that the design of the device induced unintended additional loads on the lower-limb joints, potentially increasing the risk of musculoskeletal disorders. In addition, this study identified potential design improvements to mitigate these effects. Overall, this study offers a detailed biomechanical analysis of mechanical impacts on the body and thus supports efficient improvements to wearable chair design.

## Figures and Tables

**Table 1:** Gait parameters (mean ± SD) for walking with and without the wearable chair.

	Without the Wearable Chair	With the Wearable Chair
Gait cycle * (s)	1.26±0.12	1.42±0.14
Stride length ** (m)	1.40±0.08	1.32±0.08
Walking speed ** (m)	1.12±0.11	0.94±0.10

* Statistically significant differences between two conditions are indicated by p<0.05; ** Statistically significant differences between two conditions are indicated by p<0.01.

## Data Availability

Data are contained within the article. For further information, please contact the corresponding author.

## Abbreviations

The following abbreviations are used in this manuscript:

3D	three-dimensional
DOF	degree of freedom
GRF	Ggound reaction force
ISB	International Society of Biomechanics
SD	standard deviation
CMC	coefficient of multiple correlation

## Appendix A. Transformation of External Forces and Moments

External forces Fextsseg=[Fext_xsseg,Fext_ysseg,Fext_zsseg]T and moments Mextsseg=[Mext_xsseg,Mext_ysseg,Mext_zsseg]T for the *s*-th segment in the segment coordinate system were computed from measurements Fextssen and Mextssen:

(A1) Fextsseg=RssensegFextssen

(A2) Mextsseg=rsensorsseg×Fextsseg+RssensegFextssen

where Rssenseg is the rotation matrix between the sensor and segment coordinate systems, and rsensorsseg is the position vector from the segment origin to the sensor. If a transformation to the global coordinate system is required, replace Rssenseg with Rssenglo and rsensorsseg with rsensorsglo in Equations (A1) and (A2).

## References

[1] Gell N., Werner R.A., Hartigan A., Wiggermann N., Keyserling W.M. (2011). Risk factors for lower extremity fatigue among assembly plant workers. Am. J. Ind. Med..

[2] Jin X., Dong Y., Wang F., Jiang P., Zhang Z., He L., Forsman M., Yang L. (2022). Prevalence and associated factors of lower extremity musculoskeletal disorders among manufacturing workers: A cross-sectional study in China. BMJ Open.

[3] Kong Y.-K., Park C.-W., Cho M.-U., Kim S.-Y., Kim M.-J., Hyun D.J., Bae K., Choi J.K., Ko S.M., Choi K.-H. (2021). Guidelines for working heights of the lower-limb exoskeleton (CEX) based on ergonomic evaluations. Int. J. Environ. Res. Public Health.

[4] Moore S.M., TormaKrajewski J., Steiner L.J. (2011). Practical Demonstrations of Ergonomic Principles.

[5] de Looze M.P., Bosch T., Krause F., Stadler K.S., O’Sullivan L.W. (2015). Exoskeletons for industrial application and their potential effects on physical workload. Ergonomics.

[6] Theurel J., Desbrosses K. (2019). Occupational exoskeletons: Overview of their benefits and limitations in preventing work-related musculoskeletal disorders. IISE Trans. Occup. Ergon. Hum. Factors.

[7] Ashta G., Finco S., Battini D., Persona A. (2023). Passive exoskeletons to enhance workforce sustainability: Literature review and future research agenda. Sustainability.

[8] Gonsalves N., Akanmu A., Shojaei A., Agee P. (2024). Factors influencing the adoption of passive exoskeletons in the construction industry: Industry perspectives. Int. J. Ind. Ergon..

[9] Yan Z., Han B., Du Z., Huang T., Bai O., Peng A. (2021). Development and testing of a wearable passive lower-limb support exoskeleton to support industrial workers. Biocybern. Biomed. Eng..

[10] Haraguchi N., Hase K. (2023). Biomechanical analysis based on a full-body musculoskeletal model for evaluating the effect of a passive lower-limb assistive device on lumbar load. J. Biomech. Sci. Eng..

[11] noonee AG Chairless Chair—Wearable Posture Support Device. https://www.noonee.com/.

[12] Li L., Chen Z., Hong R., Qu Y., Gao X., Wang X. (2025). Research status and development trend of lower-limb squat-assistant wearable devices. Biomimetics.

[13] Luger T., Seibt R., Cobb T.J., Rieger M.A., Steinhilber B. (2019). Influence of a passive lower-limb exoskeleton during simulated industrial work tasks on physical load, upper-body posture, postural control and discomfort. Appl. Ergon..

[14] Wang W., Liang X., Li L., Gong L., Deng L., Hu X., Zhang S., Sun S. (2024). Wearable chairless exoskeleton capable of flexible support to relieve muscle fatigue for industrial workers. Smart Mater. Struct..

[15] Baltrusch S.J., van Dieën J.H., van Bennekom C.A.M., Houdijk H. (2018). The effect of a passive trunk exoskeleton on functional performance in healthy individuals. Appl. Ergon..

[16] Steinhilber B., Seibt R., Rieger M.A., Luger T. (2022). Postural control when using an industrial lower-limb exoskeleton: Impact of reaching for a working tool and external perturbation. Hum. Factors.

[17] Li Y.Y., Gan J. (2023). Effect of wearable chair on gait, balance, and discomfort of new users during level walking with anterior loads. J. Saf. Res..

[18] Greggi C., Visconti V.V., Albanese M., Gasperini B., Chiavoghilefu A., Prezioso C., Persechino B., Iavicoli S., Gasbarra E., Iundusi R. (2024). Work-related musculoskeletal disorders: A systematic review and meta-analysis. J. Clin. Med..

[19] Kim U., Lim J., Park Y., Bae Y. (2025). Predicting fall risk through step width variability at increased gait speed in community-dwelling older adults. Sci. Rep..

[20] Davis R.B., Ounpuu S., Tyburski D., Gage J.R. (1991). A gait analysis data collection and reduction technique. Hum. Mov. Sci..

[21] Winter D.A. (2009). Biomechanics and Motor Control of Human Movement.

[22] Haraguchi N., Hase K. (2024). Multibody model with foot-deformation approach for estimating ground reaction forces and moments and joint torques during level walking through optical motion capture without optimization techniques. Sensors.

[23] Wang S., Pitts J., Purohit R., Shah H. (2025). The influence of motion data low-pass filtering methods in machine-learning models. Appl. Sci..

[24] Horsak B., Slijepcevic D., Raberger A.-M., Schwab C., Worisch M., Zeppelzauer M. (2020). GaitRec, a large-scale ground reaction force dataset of healthy and impaired gait. Sci. Data.

[25] Wang S., Cai Y., Hase K., Uchida K., Kondo D., Saito T., Ota S. (2022). Estimation of knee joint angles during gait cycle using inertial measurement unit sensors: A method of sensors-to-clinical bone calibration on the lower-limb skeletal model. J. Biomech. Sci. Eng..

[26] Wu G., Siegler S., Allard P., Kirtley C., Leardini A., Rosenbaum D., Whittle M., D’Lima D.D., Cristofolini L., Witte H. (2002). ISB recommendation on definitions of joint coordinate system of various joints for the reporting of human joint motion—Part I: Ankle, hip, and spine. J. Biomech..

[27] Wu G., van der Helm F.C.T., Veeger H.E.J., Makhsous M., Van Roy P., Anglin C., Nagels J., Karduna A.R., McQuade K., Wang X. (2005). ISB recommendation on definitions of joint coordinate systems of various joints for the reporting of human joint motion—Part II: Shoulder, elbow, wrist and hand. J. Biomech..

[28] Derrick T.R., van den Bogert A.J., Cereatti A., Dumas R., Fantozzi S., Leardini A. (2020). ISB recommendations on the reporting of intersegmental forces and moments during human motion analysis. J. Biomech..

[29] Hof A.L. (1996). Scaling gait data to body size. Gait Posture.

[30] Teng H.L., Calixto N.E., MacLeod T.D., Nardo L., Link T.M., Majumdar S., Souza R.B. (2016). Associations between patellofemoral-joint cartilage *T*_1_*ρ* and *T*_2_ and knee-flexion moment and impulse during gait in individuals with and without patellofemoral joint osteoarthritis. Osteoarthr. Cartil..

[31] Gill N., O’Leary T., Roberts A., Liu A., Roerdink M., Greeves J., Jones R. (2023). Enforcing walking speed and step-length affects joint kinematics and kinetics in male and female healthy adults. Gait Posture.

[32] van Hooren B., Hirsch S.M., Meijer K. (2023). A comparison of five methods to normalize joint moments during running. Gait Posture.

[33] Kadaba M.P., Ramakrishnan H.K., Wootten M.E., Gainey J., Gorton G., Cochran G.V.B. (1989). Repeatability of kinematic, kinetic, and electromyographic data in normal adult gait. J. Orthop. Res..

[34] Kazerooni H., Steger R., Huang L. (2006). Hybrid control of the Berkeley lower extremity exoskeleton (BLEEX). Int. J. Robot. Res..

[35] Duong M.K., Cheng H., Tran H.T., Jing Q. (2016). Minimizing human-exoskeleton interaction force using compensation for dynamic uncertainty error with adaptive RBF network. J. Intell. Robot. Syst..

[36] Sanchez-Villamañan M.D.C., Gonzalez-Vargas J., Torricelli D., Moreno J.C., Pons J.L. (2019). Compliant lower limb exoskeletons: A comprehensive review on mechanical design principles. J. Neuroeng. Rehabil..

[37] Winter D.A. (1983). Energy generation and absorption at the ankle and knee during fast, natural, and slow cadences. Clin. Orthop. Relat. Res..

[38] Neptune R.R., Kautz S.A., Zajac F.E. (2001). Contributions of the individual ankle plantar flexors to support, forward progression and swing initiation during walking. J. Biomech..

[39] Lee S.K. (2013). The effect of a vertical load on gluteus medius activity and gait characteristics during walking. J. Phys. Ther. Sci..

[40] Hwang J.W., Lee S.K., Park J.S., Ahn S.H., Lee K.J., Lee S.J. (2017). The effects of ankle weight loading on the walking factors of adults without symptoms. J. Exerc. Rehabil..

[41] Bedi A., Warren R.F., Wojtys E.M., Oh Y.K., Ashton-Miller J.A., Oltean H., Kelly B.T. (2016). Restriction in hip internal rotation is associated with an increased risk of ACL injury. Knee Surg. Sports Traumatol. Arthrosc..

[42] Ye B., Liu G., He Z., Xu J., Pan H., Zhu H. (2024). Biomechanical mechanisms of anterior cruciate ligament injury in the jerk dip phase of clean and jerk: A case study of an injury event captured on-site. Heliyon.

[43] Fong D.T.P., Chan Y.Y., Mok K.M., Yung P.S., Chan K.M. (2009). Understanding acute ankle ligamentous sprain injury in sports. BMC Sports Sci. Med. Rehabil..

[44] Vap A.R., Schon J.M., Moatshe G., Cruz R.S., Brady A.W., Dornan G.J., Turnbull T.L., LaPrade R.F. (2017). The role of the peripheral passive rotation stabilizers of the knee with intact collateral and cruciate ligaments: A biomechanical study. Orthop. J. Sports Med..

[45] Li L., Gollhofer A., Lohrer H., Dorn-Lange N., Bonsignore G., Gehring D. (2019). Function of ankle ligaments for subtalar and talocrural joint stability during an inversion movement—An in vitro study. J. Foot Ankle Res..

[46] Shin C.S., Chaudhari A.M., Andriacchi T.P. (2009). The effect of isolated valgus moments on ACL strain during single-leg landing: A simulation study. J. Biomech..

[47] Breloff S.P., Dutta A., Dai F., Sinsel E.W., Warren C.M., Ning X., Wu J.Z. (2019). Assessing work-related risk factors for musculoskeletal knee disorders in construction roofing tasks. Appl. Ergon..

[48] Brandl C., Bender A., Schmachtenberg T., Dymke J., Damm P. (2024). Comparing risk assessment methods for work-related musculoskeletal disorders with in vivo joint loads during manual materials handling. Sci. Rep..

[49] Ghai S., Ghai I. (2019). Virtual reality enhances gait in cerebral palsy: A training dose-response meta-analysis. Front. Neurol..

[50] Ghai S., Ghai I., Lamontagne A. (2020). Virtual reality training enhances gait poststroke: A systematic review and meta-analysis. Ann. N. Y. Acad. Sci..

[51] Ghai S., Ghai I., Schmitz G., Effenberg A.O. (2018). Effect of rhythmic auditory cueing on parkinsonian gait: A systematic review and meta-analysis. Sci. Rep..

[52] Koussou A., Desailly E., Dumas R. (2021). Contribution of passive moments to inter-segmental moments during gait: A systematic review. J. Biomech..

